# Alendronate overdose/intoxication: Suicidal attempt in a case report

**DOI:** 10.1192/j.eurpsy.2021.2187

**Published:** 2021-08-13

**Authors:** T. Gutiérrez Higueras, F. Calera Cortés, S. Vicent Forés, S. Sainz De La Cuesta Alonso

**Affiliations:** Clinical Unit Of Mental Health., Reina Sofia University Hospital., Córdoba., Spain

**Keywords:** alendronate, Suicide, Intoxication, Treatment

## Abstract

**Introduction:**

Alendronate is a nitrogen-containing biphosphonate that inhibits osteoclastic bone resorption. Lethal dose (LD50) was aproximately 626mg/kg in male rats, and 552mg/kg in female. Signs and Symptoms of overdose clammy skin, CNS depression, dysphagia, hiccups, miosis, respiratory depression, seizures and wheezing. Supportive therapy and monitor of urine flow, calcium and phsophorous level is essential for the management of voluntary overdose.

**Objectives:**

To present the case of a 76-year-old woman who made a suicide attempt by ingestion of 8 tablets of 70 mg of alendronate.To describe the treatment of alendronate poisoning and the follow-up parameters for the control of complications.

**Methods:**

Clinical case presentation through retrospective review of clinical notes and non-systematic literature review.

**Results:**

A 76-year-old woman was taken to the emergency department after voluntarily ingesting 8 alendronate tablets (70 mg per tablet) 1 hour ago reporting “suicidal thoughts”. After clinical evaluation, gastric lavage, administration of activated charcoal, and IV ranitidine were used. After 24-hour observation and after psychiatric evaluation, the patient was discharged.

**Conclusions:**

Hypocalcaemia, hypophosphataemia and upper gastrointestinal adverse reactions, such as upset stomach, heartburn, oesophagitis, gastritis, or ulcer, may result from oral overdose. In case of overdose with alendronate, milk or antacids should be given to bind alendronate. Giving milk or antacids, to bind the bisphosphonate and minimize absorption, has been suggested for oral overdose. Due to the risk of esophageal irritation, vomiting should not be induced and the patient should remain fully upright. For decontamination is recomended activated charcoal and gastric lavage.

**Disclosure:**

No significant relationships.

